# Editorial: Androgen receptors in male and female reproduction

**DOI:** 10.3389/fendo.2025.1638220

**Published:** 2025-06-18

**Authors:** Federica Barbagallo, Settimio D’Andrea

**Affiliations:** ^1^ Department of Clinical and Experimental Medicine, University of Catania, Catania, Italy; ^2^ Endocrinology Outpatient Clinic, Department “Area Peligno-Sangrina”, ASL 1 Abruzzo, L’Aquila, Italy

**Keywords:** androgen, androgen recepter, male reproduction, female reproduction, PCOS (polycystic ovarian syndrome), reproductive tract

Androgen actions are mediated through the androgen receptor (AR), a member of the nuclear receptor super family. The AR gene maps on X chromosome, locus Xq11-Xq12 and consists of eight exons and seven introns. The AR gene encodes a 110 kDa protein consisting of 919 amino acids. It can be structurally divided into four domains: the N-terminal domain (NTD), which is responsible for the transcriptional activation; the DNA-binding domain (DBD), which mediates gene-specific binding; the hinge region, which regulates nuclear localization and stability; and the ligand-binding domain (LBD), which binds to androgens. While traditionally associated with male reproductive function, a growing body of research highlights the essential role of AR in female reproductive physiology as well.

This Research Topic collected different studies that explore the complex role of AR in reproductive health. In males, AR has long been considered essential for the development of secondary sexual characteristics, spermatogenesis, and overall reproductive function. The genetic basis of androgen insensitivity syndrome (AIS), resulting from mutations in the AR gene, underscores the critical role of AR in male sexual differentiation and reproduction.

According to the severity of AR resistance to androgens, AIS is classified as complete AIS (CAIS), partial AIS (PAIS), and mild AIS (MAIS), giving rise to a wide clinical spectrum (1). CAIS is characterized by complete resistance to the effects of androgens and it was first described by Morris in 1953. Patients with CAIS have a female phenotype with a 46,XY karyotype. With the widespread use of modern molecular biology techniques and genetic testing, over 1,000 different mutations in the AR gene have now been reported, with new mutation sites continuously emerging (2).

Wang et al. reported the case of a phenotypic female newborn presenting with esophageal atresia and elevated bilirubin levels. Further investigations revealed testicular tissue, a 46,XY karyotype, and a novel exon 1 deletion [amino acid variant-(NM 000044)] in the AR gene identified by whole-exome sequencing, leading to a diagnosis of CAIS. This case highlights the diagnostic challenge in patients with normal female external genitalia and expands the known mutation spectrum of CAIS.

Recent studies have shed new light on the broader implications of AR in male fertility. For instance, alterations in AR signaling can influence spermatogenesis, impacting both the quantity and quality of sperm production. Furthermore, emerging evidence points to the importance of AR in the regulation of male reproductive tract development, suggesting that the receptor may have more extensive roles than previously appreciated. These insights are essential for understanding male infertility, which often has underlying molecular defects in androgen receptor signaling (3).

While much of the early research on AR focused on its role in males, it is increasingly clear that AR also exerts significant effects on female reproductive health. The presence of AR in ovarian granulosa cells, theca cells, and even the endometrial lining highlights the receptor’s broad involvement in female fertility. In particular, AR is implicated in the regulation of folliculogenesis, ovulation, and the maturation of oocytes. Recent studies have demonstrated that disruptions in AR signaling can lead to ovarian dysfunction, a phenomenon observed in conditions such as PCOS (4). PCOS, one of the most common endocrine disorders in women, is often characterized by hyperandrogenism (HA). Understanding how AR contributes to the pathophysiology of PCOS is crucial for developing new diagnostic and therapeutic strategies. Research on the interaction between androgens and AR in the ovaries has revealed a complex network of signaling pathways that regulate follicular development and steroidogenesis. Furthermore, these studies suggest that AR may be a potential target for therapeutic intervention, with the potential to alleviate symptoms of PCOS and improve fertility outcomes (5). Gao et al. conducted an integrative bioinformatics analysis to explore the molecular mechanisms linking HA to immune dysfunction and impaired fertility outcomes in PCOS. Through weighted gene co-expression network analysis (WGCNA) and protein-protein interaction (PPI) network construction, the study highlighted immune-related gene modules and inflammatory pathways associated with hyperandrogenic PCOS (HA PCOS). Key findings included the identification of DAPK2 (*death-associated protein kinase 2*), as a critical gene linked to granulosa cell apoptosis, immune dysregulation, and recurrent implantation failure (RIF) Moreover, different genes were identified as co-expressed in HA-PCOS and RIF, pointing to dysregulation in corticosteroid metabolism, bone maturation, and immune responses. These results suggest that androgen excess contributes to abnormal granulosa cell function, placental morphology, and endometrial receptivity, thereby increasing the risk of pregnancy complications and implantation failure.

While HA in PCOS is associated with impaired granulosa cell function and disrupted folliculogenesis, several studies, including that of Li et al., have reported a positive correlation between androgens levels and elevated levels of anti-Müllerian hormone (AMH), which is widely used as a biomarker for ovarian reserve. This underscores the need for further research into the molecular mechanisms that govern tissue-specific AR activation and its impact on fertility (6).

Recent advances in genetic tools have allowed researchers to explore the function of AR in greater detail. Animal models, particularly those involving androgen receptor knockout (ARKO) mice, have been instrumental in elucidating the role of AR in both male and female reproductive physiology. Interestingly, female ARKO mice exhibit reproductive defects that challenge our understanding of AR’s role in female fertility. These mice demonstrate impaired folliculogenesis and an early onset of ovarian failure, underscoring the importance of AR in maintaining ovarian function. Moreover, research on ARKO mice has revealed differences in the way androgens influence reproductive tissues in males and females, suggesting that the effects of AR signaling are context-dependent. Feng et al. demonstrated that female LivARKO mice, which lack hepatic androgen receptor (AR), exhibit normal pubertal onset, estrous cyclicity, and fertility. Upon dihydrotestosterone (DHT) treatment develop disrupted estrous cycles, reduced corpora lutea, and infertility. These findings indicate that hepatic AR is not required for the manifestation of HA-induced reproductive dysfunction. Furthermore, reproductive dysfunction in the DHT-treated LivARKO lean females with normal glucose homeostasis indicates that androgen-induced reproductive dysfunction is independent from metabolic dysfunction in lean conditions.

The identification of AR as a key player in reproductive health opens up exciting possibilities for the development of novel therapies. For example, selective modulators of AR could offer new treatment options for conditions such as PCOS, where the receptor’s activity may be overactive. Conversely, AR antagonists could be used to mitigate the effects of excessive androgenic signaling in disorders like endometriosis or certain types of infertility. Therapeutic approaches targeting AR could also extend to male infertility, particularly in cases where AR mutations or dysfunction are involved. However, the challenge lies in the tissue-specific nature of AR signaling, as targeting the receptor without affecting other important systems could prove difficult. Therefore, future research will need to focus on the development of more refined AR modulators that can act selectively on specific tissues or organ. As previously mentioned, HA is a leading cause of dysfunction in ovarian granulosa cells of PCOS patients. Chen et al. reported that curcumin exerts protective effects against DHT-induced damage by enhancing estrogen synthesis and inhibiting AR activity, thereby restoring cell viability and correcting lipid metabolism disruptions caused by elevated androgen levels.

This Research Topic underscores the complexity of AR signaling in both male and female reproductive function. The contributions collected here shed light on the multifaceted roles of AR in reproductive tissues, highlighting both shared features and differences across sexes. We truly hope that some of these studies will help address open questions in the field, stimulate further *ad hoc* research, and ultimately contribute to a more refined understanding of AR signaling. In turn, this could pave the way for future advances in diagnostic and therapeutic strategies for a wide range of reproductive disorders.

## References

[1] Matsumoto T Kawano H Shiina H Sato T Kato S. Androgen receptor functions in male and female reproduction. Reprod Med Biol. (2007) 6(1):11–7. doi: 10.1111/j.1447-0578.2007.00159.x PMC5904580 29699261

[2] Barbagallo F Cannarella R Bertelli M Crafa A La Vignera S Condorelli RA. Complete androgen insensitivity syndrome: from the relevance of an accurate genetic diagnosis to the challenge of clinical management. A Case Rep Medicina (Kaunas). (2021) 57:1142. doi: 10.3390/medicina57111142 PMC8624150 34833359

[3] Matsumoto T Sakari M Okada M Yokoyama A Takahashi S Kouzmenko A. The androgen receptor in health and disease. Annu Rev Physiol. (2013) 75:201–24. doi: 10.1146/annurev-physiol-030212-183656 23157556

[4] Matsumoto T Shiina H Kawano H Sato T Kato S. Androgen receptor functions in male and female physiology. J Steroid Biochem Mol Biol. (2008) 109(3–5):236–41. doi: 10.1016/j.jsbmb.2008.03.023 18434134

[5] Tsai YR Liao YN Kang HY. Current advances in cellular approaches for pathophysiology and treatment of polycystic ovary syndrome. Cells. (2023) 12(17):2189. doi: 10.3390/cells12172189 37681921 PMC10487183

[6] Davis SR Wahlin-Jacobsen S. Testosterone in women: the clinical significance. Lancet Diabetes Endocrinol. (2015) 3:980–92. doi: 10.1016/S2213-8587(15)00345-2 26358173

